# Bilirubin Induces Pain Desensitization in Cholestasis by Activating 5-Hydroxytryptamine 3A Receptor in Spinal Cord

**DOI:** 10.3389/fcell.2021.605855

**Published:** 2021-04-01

**Authors:** Erliang Kong, Hongqian Wang, Xiaoqiang Wang, Yan Zhang, Jinmin Zhang, Weifeng Yu, Xudong Feng, Yuming Sun, Feixiang Wu

**Affiliations:** ^1^Department of Anesthesiology, Shanghai Eastern Hepatobiliary Surgery Hospital, Naval Medical University, Shanghai, China; ^2^Department of Anesthesiology, The 988th Hospital of Joint Logistic Support Force of PLA, Zhengzhou, China; ^3^Department of Anesthesiology, Shandong Provincial Hospital Affiliated to Shandong First Medical University, Jinan, China; ^4^Department of Anesthesiology, Renji Hospital, Shanghai Jiao Tong University School of Medicine, Shanghai, China; ^5^Department of Anesthesiology, Zhejiang Province Zhoushan Hospital, Zhoushan, China; ^6^Department of Critical Care Medicine, Shanghai Eastern Hepatobiliary Surgery Hospital, Naval Medical University, Shanghai, China

**Keywords:** bilirubin, cholestasis, 5-HT_3*A*_ receptor, pain desensitization, GABA

## Abstract

**Background:**

Cholestasis patients often suffer from pain desensitization, resulting in serious complications in perioperative period. This study was aim to investigate the mechanism of bilirubin in cholestasis mediating pain desensitization through 5-hydroxytryptamine 3A (5-HT_3*A*_) receptor activation in spinal dorsal horn (SDH).

**Methods:**

A cholestasis model was established by bile duct ligation (BDL) in rats. Pain thresholds of rats were measured after BDL or intrathecally injecting bilirubin in the presence or absence of agonist (mCPBG) and antagonists (ondansetron, bicuculline, or CGP55845). Expression of 5-HT_3_ receptors, and the affinity and binding mode of bilirubin to 5-HT_3*A*_ receptor were determined. Effects of bilirubin on γ-aminobutyric acid (GABA) pathway and the interactions with 5-HT_3*A*_ receptor were tested.

**Results:**

Bilirubin was elevated significantly in both serum and CSF in BDL rats, accompanied with the up-regulation of pain thresholds. Both of 5-HT_3*A*_ receptor and GABA_*A*_ receptor antagonists could reverse the increased pain threshold in BDL rats. Further, 5-HT_3*A*_ and GABA_*A*_ receptor expressions were increased in BDL rats or intervention with bilirubin. Molecular docking suggested that bilirubin entered the hydrophobic pocket pre-formed in 5-HT_3*A*_ receptor with potential hydrogen bonding. Bilirubin also increased GABA concentrations in CSF and GABAergic spontaneous inhibitory postsynaptic current in spinal cord, and directly induced inward currents in HEK293 cells which were overexpressed 5-HT_3*A*_ receptor by lentivirus.

**Conclusion:**

In conclusion, bilirubin induced pain desensitization in cholestasis by activating 5-HT_3*A*_ receptor in spinal cord. The activation of 5-HT_3*A*_ receptor might regulate pain threshold by acting on the GABA pathway.

## Introduction

Cholestasis is characterized by the obstruction of intrahepatic/extrahepatic bile ducts by stones, tumors, or pancreatic diseases (Hegade et al., 2015). Patients with cholestasis show insensitivity to nociceptive stimulation, which often leads to the concealment of symptoms and delayed treatment (Tian et al., 2016). Moreover, respiratory depression and delayed recovery occur more frequently in cholestasis patients, resulting in an increased risk of death in the perioperative period. Thus, exploration of the mechanism of pain desensitization in cholestasis can provide theoretical basis and treatment targets for cholestasis.

Multiple substances such as 5-hydroxytryptamine (5-HT), endogenous opioids, and cholecystokinin participate in pain transmission in cholestasis (Hasanein and Javanmardi, 2008). The 5-HT pathway is regarded to mediate various physiologic activities by seven receptors, and the 5-HT_3_ receptors are the only ligand-gated ion channel in the 5-HT receptor family (Hannon and Hoyer, 2008). The 5-HT_3_ receptors are widely distributed in the central nervous system (CNS), especially the spinal dorsal horn (SDH). Activation of 5-HT_3_ receptors in SDH produces an anti-nociceptive response, whereas the antagonists of 5-HT_3_ receptors can reverse this effect. Blockers of the γ-aminobutyric acid (GABA) pathway can also reverse the anti-nociceptive effect caused by the 5-HT_3_ receptors, suggesting that the 5-HT_3_ receptors in SDH could regulate pain threshold through GABA pathway (Pelissier et al., 1996; Iwasaki et al., 2013; Roczniak et al., 2013; Bobinski et al., 2015). It has also been confirmed that morphine regulated pain transmission by combining with the 5-HT_3*A*_ receptor (Schaaf et al., 2014).

Bilirubin, with a molecular weight of 584, derives from senescent erythrocyte, hemoglobin, and ineffective hematopoiesis. Free bilirubin that does not bind to glucuronic acid, can damage the blood-brain barrier (BBB) and increase its permeability directly. As a small molecule, bilirubin can pass the BBB readily and be toxic to gliocyte and neurons. Bilirubin can also induce inflammation, neurodegeneration and apoptosis, which can affect cognition, memory, pain sensation, and can even cause kernicterus in newborns.

In patients with cholestasis, concentrations of bilirubin usually increase significantly in serum and cerebrospinal fluid (CSF) because indirect bilirubin (IB, also characterized by unconjugated bilirubin) can pass through the damaged BBB into the CNS (Cardoso et al., 2012; Xu et al., 2016). Admittedly, morphine can exert an analgesic role clinically by activating opioids receptors. Additionally, morphine and bilirubin also share similar spatial structure. Recent studies have suggested that morphine and bilirubin can both induce pain regulation, withdrawal reaction, and chronic itching (Meixiong et al., 2019; Shah and John, 2020). Therefore, we hypothesized that elevated bilirubin in the CNS might mediate pain desensitization in cholestasis by activating the 5-HT_3*A*_ receptor and GABA pathway in the spinal cord.

## Materials and Methods

### Animals and Drugs

This study was carried out in accordance with the International Association for the Study of Pain guidelines and the Ethics Committee of the Second Military Medical University (Shanghai, China). Adult male Sprague–Dawley rats (200–220 g) were provided by the Shanghai Experimental Animal Center of the Chinese Academy of Sciences (Shanghai, China). Rats were housed in a pathogen-free environment (room temperature of 24°C and 50% humidity) under a 12 h light-dark cycle, with food and water *ad libitum*. Animals were sacrificed before pentobarbital sodium 30 mg/kg intraperitoneal anesthetization.

Drugs used in this study were intrathecally injected through PE-10 catheter implantation (Njoo et al., 2014) in 10 μL normal saline (0.9%) mixed with: bilirubin (Sigma-Aldrich, Saint Louis, MO, United States) 250, 500, or 1000 μM; 1-(m-Chlorophenyl)-biguanide (mCPBG, 5-HT_3*A*_ receptor agonist; Sigma-Aldrich) 10 μg; ondansetron (5-HT_3*A*_ receptor antagonist; Sigma-Aldrich) 10, 30, or 50 μg; bicuculline (GABA_*A*_ receptor antagonist; Abcam, Cambridge, United Kingdom) 1 μg; and CGP55845 (GABA_*B*_ receptor antagonist; Abcam) 0.5 μg. An unconjugated bilirubin stock solution (10 mM) was prepared in 0.1 M NaOH immediately before use and the pH of the incubation medium was restored to 7.4 by addition of equal amounts of 0.1 M HCl.

### Grouping

Rats were randomly divided into sham and bile duct ligation (BDL)−1, −3, −5, and −7 groups to test changes in pain thresholds depending on the days after BDL (*n* = 6). The intrathecally administered bilirubin and that with mCPBG groups were subdivided into pre-drugs and post-drugs groups to test pain thresholds with or without ondansetron, bicuculline, or CGP55845 pretreatment for 1 h (*n* = 6). Further, the functions of the 5-HT_3*A*_ receptor and the GABA receptors were evaluated. Rats were divided into saline, ondansetron (30 μg), bicuculline (1 μg), and CGP55845 (0.5 μg) groups to measure changes of pain thresholds after intrathecal administration of antagonists only (*n* = 6). Western blot was performed in the BDL group, the intrathecally administered bilirubin group, and the spinal dorsal horn neurons (SDHNs) cultured with bilirubin group (*n* = 6) to detect changes in expression of the 5-HT_3*A*_ receptors and GABA receptors. GABA concentrations of CSF were detected in the BDL group and intrathecally administered bilirubin group (*n* = 6). Changes of GABAergic spontaneous inhibitory postsynaptic current (sIPSC) were recorded in the ACSF group, the mCPBG group, bilirubin group, the mCPBG + ondansetron group, and bilirubin + ondansetron group (*n* = 4).

### Creation of Cholestasis Rat Model

The model of cholestasis was established by BDL (Yang et al., 2015). Rats were anesthetized by administering 30 mg/kg of pentobarbital sodium intraperitoneally. After opening the abdominal cavity, the bile duct was ligated at a point proximal to the hilus and a point immediately distal to the entry of the bile duct, and transected between two ligatures. Sham operation was carried out in a similar manner without ligation or transection. Cholestasis was confirmed by serum biochemical analysis and proximal dilation of the bile duct.

### Biochemical Analyses of Liver Function Indices in Serum and CSF

The blood and CSF samples of rats were placed and centrifuged for 10 min at room temperature for 10 min, then the supernate was drawn to detect liver function indexes immediately under light protection. The automatic biochemical analyzer (Hitachi 7000) was used to detect [alanine aminotransferase (ALT), aspartate aminotransferase (AST), total bilirubin (TB), direct bilirubin (DB), IB, and total bile acids (TBAs)] in the sample.

### Measurement of Mechanical and Thermal Pain Thresholds

Mechanical and thermal pain thresholds were measured in a quiet room between 09:00 and 11:00. Rats were allowed to acclimatize to surroundings for 2 h in a set of Plexiglas^TM^ cages with wire mesh floor. Von Frey filaments (North Coast Medical, Gilroy, CA, United States) of increasing stiffness were applied to six sites distributed across the plantar surface of the rat hindpaw until the filaments bent slightly. A positive withdrawal was scored if rat showed a brisk withdrawal. Eight von Frey monofilaments (0.5, 1, 2, 4, 6, 8, 10, and 12 g) were used in testing. Pain threshold was defined as the force corresponding to a 50% withdrawal, and was determined by a Hill equation linear fitting [Origin v6.0 (MicroCal, Studio City, CA, United States)] (Xiang et al., 2008). Thermal threshold was undertaken in individual cages with a transparent acrylic floor. After acclimatization, the heating device was placed under the hindpaw at certain heat intensity and a cut-off time of 30 s to avoid tissue damage. The withdrawal latency of the hindpaw was detected thrice for each rat at an interval of 5 min, and the mean value was taken as the thermal threshold.

### Western Blotting

L4-5 vertebral segments and cultured SDHNs were collected to test the expression of related proteins. Tissues and cultured SDHNs were sonicated in ice-cold RIPA lysis buffer. Protein concentrations were measured using the bicinchoninic acid protein assay kit (Pierce, Rockford, IL, United States). Proteins were denatured by heating at 99°C for 10 min, followed by separation using 10% sodium dodecyl sulfate-polyacrylamide gel and transferred onto polyvinylidene fluoride (PVDF) membranes (Bio-Rad Laboratories, Hercules, CA, United States). PVDF membranes were blocked using 5% bovine serum albumin for 2 h, followed by incubation with primary antibodies at 4°C overnight. Finally, they were incubated with secondary antibodies for 2 h. The primary antibodies used were listed as follows: goat 5-HT_3*A*_ antibody (1:1000; Abcam), rabbit 5-HT_3*B*_ antibody (1:1000; Abcam), rabbit 5-HT_3*C*_ antibody (1:1000; Abcam), rabbit 5-HT_3*D*_ antibody (1:1000; Affinity Biologicals), rabbit 5-HT_3*E*_ antibody (1:1000; Abcam), rabbit GABA_*A*_ receptor antibody (1:1000; Affinity Biologicals), rabbit GABA_*B*_ receptor antibody (1:1000; Affinity Biologicals), mouse GAPDH antibody (1:2000; Proteintech). Proteins were detected using enhanced chemiluminescence, and densitometry was performed using Image J software.

### Immunofluorescence

L4-5 vertebral segments and cultured SDHNs were prepared for immunofluorescence to observe neuron activation and 5-HT_3*A*_ receptor expression. L4-5 vertebral segments were post-fixed in 4% paraformaldehyde and dehydrated in 30% sucrose solution. Next, transverse frozen tissue sections (thickness, 20 μm) were cut using a freezing microtome (Leica, Wetzlar, Germany). Similarly, SDHNs cultured on cover glasses were post-fixed in 4% paraformaldehyde for 30 min. Then, spinal-cord slices and cultured SDHNs were incubated with 5% goat serum for 2 h, followed by incubation with primary antibodies at 4°C. Finally, they were incubated with secondary antibodies for 2 h. The primary antibodies were listed as follows: mouse glutamic acid decarboxylase (GAD) 65 antibody (1:200; Abcam), mouse microtubule-associated protein 2 (MAP_2_) antibody (1:200; Santa Cruz Biotechnology), and mouse C-fos antibody (1:200; Santa Cruz Biotechnology). Digital images were captured with a high-resolution fluorescence microscope and merged by Photoshop^TM^ (Adobe, San Jose, CA, United States).

### Enzyme-Linked Immunosorbent Assay

GABA concentrations in CSF were tested by enzyme-linked immunosorbent assay (ELISA) to observe the effect of bilirubin on GABA release. CSF samples of rats were acquired by puncture of the occipital fonticulus. 50 μL of standards and samples were prepared in the sample diluent and eight standards (0, 1.56, 3.12, 6.25, 12.5, 25, 50, and 100 ng/mL) were established. Briefly, 50 μL of Detection A Working Solution was added immediately and incubated for 1 h at 37°C. Being aspirated and washed, each well added 100 μL of Detection B Working Solution, followed by incubation for 45min at 37°C. After aspirating and washing again, each well added 90 μL of Substrate Solution, followed by incubation for 20 min in dark at 37°C. Then, 50 μL of Stop Solution was added to each well. Finally, the optical density of each well was determined simultaneously using PlateReader AF2000 (Eppendorf, Hamburg, Germany) at 450 nm.

### Culture of SDHNs

Spinal dorsal horn neurons were taken from fetuses of late-pregnancy rats and cultured with bilirubin. Briefly, fetuses in uteruses of 20-day pregnant rats were removed into Hanks buffer after deep anesthesia (3% desflurane inhalation). Under dissecting microscope, the spinal cord was split down the midline, and each half divided longitudinally into dorsal and ventral strips. The dorsal strips of all fetuses were dissociated by trituration and placed in a tube along with 0.25% trypsin for 15 min digestion at 37°C. Then the mixture was centrifuged at 200 *g* for 4 min and rinsed several times with Hanks buffer. Cells were plated onto six-well plates coated with poly-L-lysine and cultured in Neurobasal^TM^ Growth Medium maintained at 37°C in the humidified incubator at an atmosphere of 5% CO_2_ (Langlois et al., 2010).

### Electrophysiological Testing of Spinal Cord Slices

The effect of bilirubin on GABAergic sIPSC in SDH was recorded by patch clamping. Lumbar enlargements of spinal cords were moved into ice-cold ACSF whose composition was (in mM): NaCl 117, KCl 3.6, CaCl_2_ 2.5, MgCl_2_ 1.2, NaH_2_PO_4_ 1.2, NaHCO_3_ 25, and glucose 11 with 95% O2 + 5% CO2 air and cut transversely at 400 μm. The slices were incubated for recovery at 33°C and transferred to a recording chamber with ACSF perfusion. The patch electrode, filled with pipette solution whose composition was (in mM): Cs_2_SO_4_ 110, CaCl_2_ 0.5, MgCl_2_ 2, EGTA 5, HEPES 5, TEA 5, and ATP-Mg 5, was advanced onto the surface of neurons from substantia gelatinosa, followed by cytomembrane being sucked in. sIPSC was recorded in voltage-clamp mode using MultiClamp^TM^ 700B amplifier (Molecular Devices, Silicon Valley, CA, United States) and pClamp10.2 (Axon Instruments, Union City, CA, United States) data-acquisition software under perfusion with 10 μM bilirubin, 30 μM mCPBG, or 50 μM ondansetron.

### Human 5-HT_3*A*_ Receptor Overexpression and Electrophysiological Testing of Human Embryonic Kidney 293 Cells

Lentivirus with plasmids carrying the target gene (human 5-HT_3*A*_ receptor) were prepared and integrated into host cells to acquire highly efficient human embryonic kidney (HEK293) cell lines overexpressing the human 5-HT_3*A*_ receptor (HEK293-5-HT3A cells). HEK293 cells were divided into: the blank group without lentivirus transfection; the control group with blank lentivirus transfection; and the plasmid group with successful 5-HT_3*A*_-lentivirus transfection. Currents induced by bilirubin on HEK293-5-HT_3*A*_ cells were recorded. HEK293 cells were transferred to round glass plates and cultured for 24 h with individual density. Then, the glass plates were placed in a recording groove with extracellular fluid perfusion whose composition was (in mM): NaCl 140, KCl 4.7, CaCl_2_ 2, MgCl_2_ 1, HEPES 10, and glucose 11. An electrode containing pipette solution, whose composition was (in mM): CsCl 110, CaCl_2_ 1, MgCl_2_ 1, EGTA 10, HEPES 10, TEA-Cl 25, and ATP-Na_2_ 2, was moved to the cell surface, followed by cytomembrane being sucked in. The inward currents generated by drug perfusion were recorded at −50 mV using the MultiClamp 700B amplifier and pClamp10.2 data-acquisition software.

### Radioligand Binding Assay

The affinity of bilirubin and the 5-HT_3*A*_ receptor was measured by radioligand binding assay. 5-HT (non-specific agonist of 5-HT_3*A*_ receptor) was used as a positive control and 8-OH-DPAT (5-HT_1*A*_ receptor agonist) as a negative control. Glycodesoxycholic acid (GDCA, one kind of bile acid), which increased in cholestasis, was also tested. HEK293-5-HT3A cells were cultured, trypsinized, and collected. After centrifugation, supernatants were collected and protein concentrations of membrane samples were determined by bicinchoninic acid protein assay kit. Membrane samples were diluted to 10 doses at five-fold serial dilution in dimethylsulfoxide (DMSO). Then, [3H]-Granisetron was transferred into wells. Meanwhile, the filters within Unifilter-96 GF/C filter plates (PerkinElmer, Waltham, MA, United States) were soaked in 0.5% polyethyleneimine for 30 min. When binding assays were completed, the contents of the binding assay were vacuumed through the filters. After being washed and dried, the bottom of the filter-plate wells was sealed using Unifilter-96 Backing Seal Tape (PerkinElmer), followed by the addition of 50 μL of Microscint^TM^ 20 Cocktail (PerkinElmer) and sealing of the top of the filter plates with TopSeal-A Sealing Film (PerkinElmer). Finally, [3H] trapped on filters was counted using a TopCount NXT HTS Reader (PerkinElmer). Data were analyzed using GraphPad Prism 5 (San Diego, CA, United States). Inhibition (%) was calculated using the following equation:

Inhibition(%)=100×[1-(rawvalueofthesample-

meanvalueforlowcontrol)/(meanvalueforhighcontrol-

meanvalueforlowcontrol)]

The half-maximal inhibitory concentration (IC50) was determined using four-parameter logistic non-linear regression analysis employing the following equation:

Y=Bottom+(Top-Bottom)/(1+10∧[(logIC50-X)×(Hillslope)])

where Y is the inhibition rate and X is the log concentration of the sample.

### Homologous Modeling

Homologous modeling uses large numbers of known three-dimensional protein structures to predict unknown protein structures. Proteins are characterized to belong to the same family if they share more than 40% similarity in homology. Proteins with known structures can be searched as candidate templates if they possess a mostly similar sequence to the target protein. Then, alignment is undertaken between the target sequence and candidate template. Multiple template combinations are selected as the final template depending on the sequence identity. The atoms that are exactly the same as the template sequence are copied completely, whereas only skeleton atoms are copied from the similar template. Side-chain coordinates and missing sequences are supplemented in a loop database. Finally, energy optimization under specific potential field parameters is done to obtain the final model.

### Molecular Docking

Molecular docking is based on the lock-key relationship to simulate the interaction between ligands and receptors. Interactions of bilirubin and the 5-HT_3*A*_ receptor were established and optimized by AutoDock^TM^. The structure of bilirubin ligand was drawn and energy was optimized using Chenmidraw 11.0 software, and Autodock^TM^ 4.0 was used to conduct the hydrogenation, calculate the charge, and combine non-polar hydrogen. The result acquired from homologous modeling was also used to conduct the hydrogenation, calculate the charge, and combine non-polar hydrogen in Autodock^TM^ 4.0. The ligand and receptor files were opened in Autogrid software, and a 60 × 60 × 60 box centered on the active sites of the protein was established. Finally, the ligand and receptor files were calculated in Autodock^TM^ 4.0 software for 30 times in a flexible molecular docking method to acquire the final results.

### Statistical Analyses

Statistical analyses were carried out using the IBM SPSS Statistics 23.0 (SPSS Inc., Armonk, NY, United States). Quantitative data are presented as the mean ± SD. Two-way repeated-measure ANOVA was used to compare withdrawal thresholds among different groups and at different time points. The relative gray value of western blotting and immunofluorescence was tested using one-way ANOVA followed by Dunnett’s *t*-test. Electrophysiological results were analyzed using Clampfit (Axon Instruments). *P*-values < 0.05 were considered statistically significant.

## Results

### Bilirubin Increased Pain Threshold of Cholestasis Rats by Activating the 5-HT_3*A*_ Receptor

**FIGURE 1:**
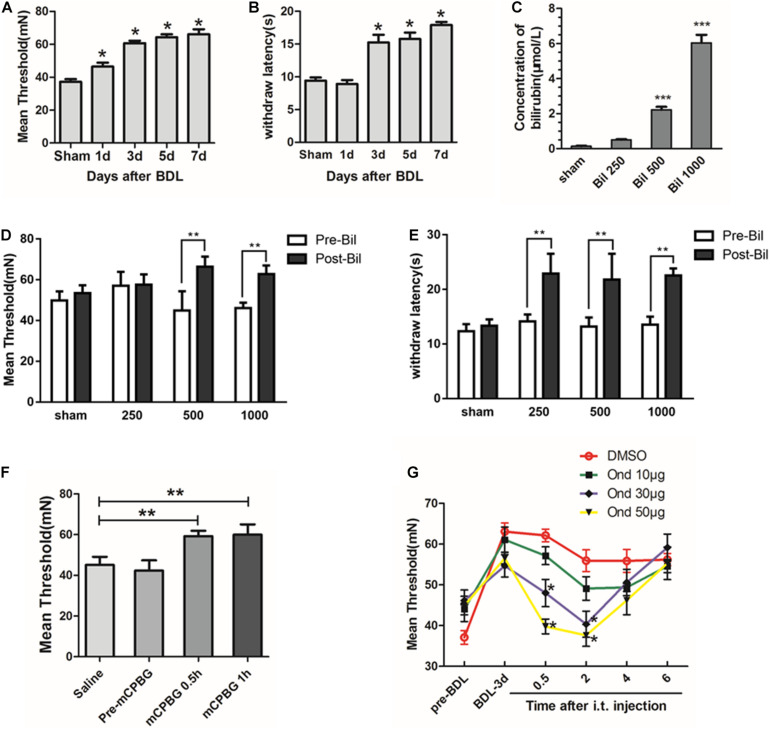
Bilirubin increased pain threshold of cholestasis rats by activating the 5-HT_3*A*_ receptor. **(A)** Mechanical pain threshold increased significantly in BDL rats on days 1, 3, 5, and 7 compared with sham group. **(B)** Thermal pain threshold increased significantly in BDL rats on days 3, 5, and 7 compared with sham group. **(C)** Indirect bilirubin level in CSF increased 20 min after intrathecal administration of bilirubin. **(D)** Mechanical pain threshold increased by 48 and 36% at 500 and 1000 μM after administrating bilirubin intrathecally, respectively. **(E)** Intrathecal administration of bilirubin increased the thermal withdrawal latency by 62, 65.2, and 66.9% at 250, 500, and 1000 μM, respectively. **(F)** Intrathecal administration of mCPBG—5-HT_3*A*_ receptor agonist—increased mechanical threshold in normal rats compared with intrathecal administration of saline. **(G)** Intrathecal administration of ondansetron—a 5-HT_3*A*_ receptor antagonist—reversed the increase of mechanical pain threshold in BDL-3-day rats in a dose-dependent manner. *P < 0.05; **P < 0.01; ***P < 0.001.

Biochemical analyses of serum showed that liver function indices (ALT, AST, TB, DB, IB, and TBAs) increased significantly in a time-dependent manner (Supplementary Table 1), suggesting that cholestasis model was created successfully. However, only IB levels in CSF increased significantly 3 days after BDL and was maintained stably (1.15 ± 0.3 μmol/L) without changes in TBAs (Supplementary Table 2), suggesting that indirect bilirubin could pass the BBB in BDL rats. Then, by testing pain thresholds, we found that mechanical and thermal pain thresholds increased significantly in BDL rats on days 3, 5, and 7 compared with the sham group (*P* < 0.05, Figures 1A,B). For further detecting the effects of bilirubin on pain thresholds in the spinal cord, different concentrations of bilirubin were intrathecally administrated into normal rats. Results showed that indirect bilirubin levels in CSF increased significantly 20 min after intrathecal administration (Figure 1C). At the same time, mechanical and thermal thresholds also increased significantly than the sham group (Figures 1D,E).

To explore whether the 5-HT_3*A*_ receptor participates in the regulation of pain thresholds in the spinal cord, mCPBG (5-HT_3*A*_ receptor agonist) was intrathecally administrated into normal rats. Results suggested that the mechanical pain threshold was significantly increased after mCPBG administration (Figure 1F). Then, ondansetron (5-HT_3*A*_ receptor antagonist) was used in BDL rats, and the results showed that ondansetron could reverse the up-regulation of pain threshold in BDL-3-day rats in a dose-dependent manner (Figure 1G). It suggested that bilirubin might induce pain desensitization by activating the 5-HT_3*A*_ receptor in cholestasis rats.

### Bilirubin Increased 5-HT_3*A*_ Receptor Expression and Neuron Activities in the Spinal Cord

**FIGURE 2:**
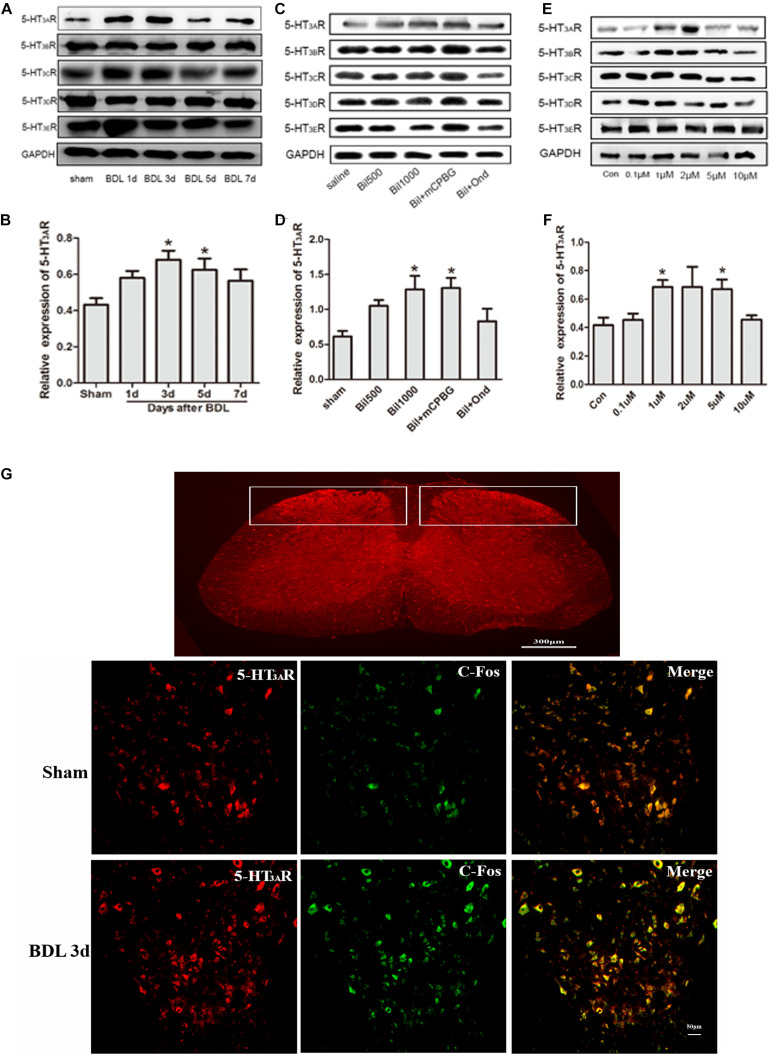
Bilirubin increased 5-HT_3*A*_ receptor expression and neuron activities in the spinal cord. **(A)** 5-HT_3*A*_ receptor expression increased gradually in spinal cord enlargement of BDL rats, whereas expression of 5-HT_3*B*_, 5-HT_3*C*_, 5-HT_3*D*_, and 5-HT_3*E*_ receptors did not change significantly. **(B)** The quantitative analysis of 5-HT_3*A*_ receptor expression in BDL rats. **(C)** Intrathecal administration of bilirubin increased 5-HT_3*A*_ receptor expression without changing other subtypes. **(D)** The quantitative analysis of 5-HT_3*A*_ receptor expression after intrathecal administration of bilirubin in normal rats. **(E)** SDHNs cultured with bilirubin showed increased 5-HT_3*A*_ receptor expression without changing other subtypes. **(F)** The quantitative analysis of 5-HT_3*A*_ receptor expression in SDHNs. **(G)** Immunofluorescence showed that 5-HT_3*A*_ receptor expression increased in laminas I and II in the spinal cords of BDL rats. The increased C-Fos protein (marker of neuron activity) expression indicated that neuron activities were also enhanced in the spinal cords of BDL rats. *P < 0.05.

Expressions of 5-HT_3_ receptors in the spinal cords of BDL rats were determined by western blotting, and the results showed that only the 5-HT_3*A*_ receptor expression increased significantly, especially in BDL-3d and BDL-5d, whereas expressions of 5-HT_3*B*_, 5-HT_3*C*_, 5-HT_3*D*_, and 5-HT_3*E*_ receptors did not change (Figures 2A,B). In addition, the up-regulation of 5-HT_3*A*_ receptor was observed in normal rats by intrathecally administrating bilirubin without changes in other subtypes (Figures 2C,D). Ondansetron could reverse the increasing of 5-HT_3*A*_ receptor expression induced by bilirubin-treated rats. Similarly, SDHNs cultured in different concentrations of bilirubin could also increase 5-HT_3*A*_ receptor expression, but not other subtypes (Figures 2E,F).

To understand the interaction between the activation of the 5-HT_3*A*_ receptor and neuron activity in the spinal cord, immunofluorescence of SDH was carried out. Results showed that the expression of the 5-HT_3*A*_ receptor increased in laminae I and II of the SDH in BDL rats (Figure 2G). Additionally, increased C-Fos protein (marker of neuron activity) expression in SDH indicated that more neurons were activated in BDL rats (Figure 2G). It suggested that the up-regulation of the 5-HT_3*A*_ receptor might play a key role in the enhancement of neuron activity in BDL rats.

### Bilirubin Directly Activated 5-HT_3*A*_ Receptor With Low Affinity by the Formation of Hydrogen Bonds

**FIGURE 3:**
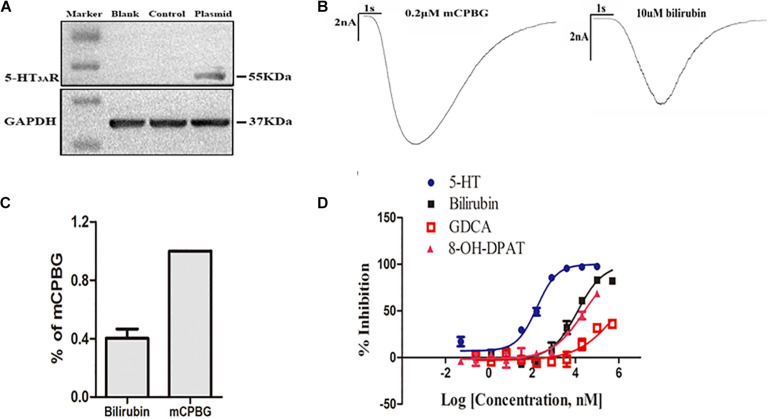
Bilirubin directly activated the 5-HT_3*A*_ receptor with low affinity. **(A)** Western blot analysis showed the establishment of HEK293 cells overexpressing the human 5-HT_3*A*_ receptor was successful. **(B)** Both mCPBG and bilirubin could induce obvious inward currents in HEK293 cells. **(C)** Normalized current intensity of HEK293 cells induced by bilirubin and mCPBG. **(D)** The dose-response curve showed that the affinity of bilirubin for the 5-HT_3*A*_ receptor fell in between 5-HT and 8-OH-DPAT.

**FIGURE 4:**
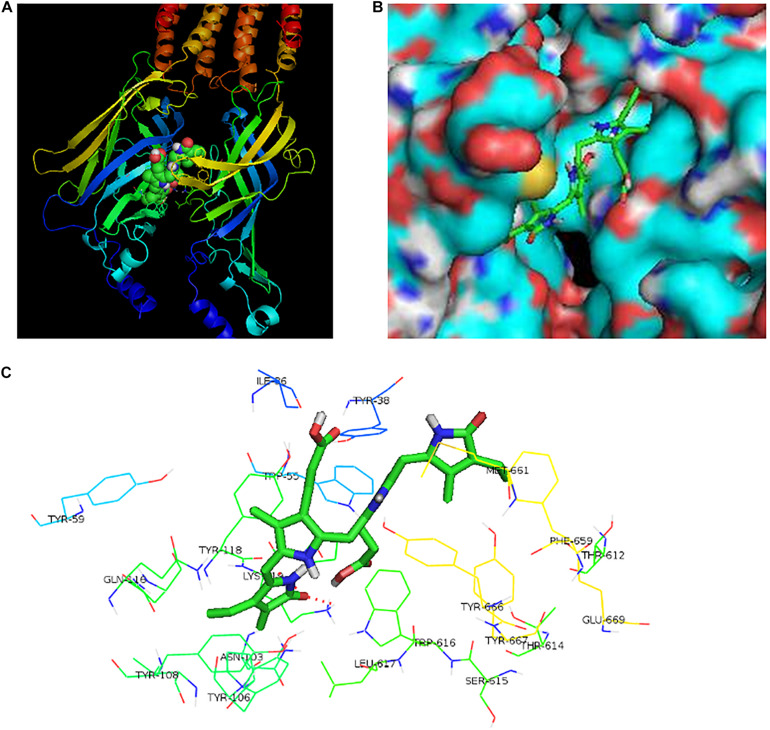
The potential binding sites of bilirubin and the 5-HT_3*A*_ receptor. **(A)** Bilirubin combined with the spatial structure of 5-HT_3*A*_ receptor. **(B)** Bilirubin entered the hydrophobic gap formed by 5-HT_3*A*_ receptor subunits with benign complementarity. **(C)** Through calculation, bilirubin was able to bond to multiple amino acid residues of the 5-HT_3*A*_ receptor with hydrogen bond formation. The binding sites of bilirubin and the 5-HT_3*A*_ receptor were MET661, LYS 119, TYR118, ILE86, TYR38, and ASN103. Hydrogen bonds were found between TYR38, LYS119 and two carbonyls of bilirubin.

To further find the direct evidence that bilirubin could activate the 5-HT_3*A*_ receptor, we overexpressed the human 5-HT_3*A*_ receptor in HEK293 cells by lentivirus. Western blotting demonstrated that transfected HEK293 cells successfully overexpressed the 5-HT_3*A*_ receptor (Figure 3A), and both mCPBG and bilirubin (10 μM) could successfully induce inward currents on HEK293 cells (Figure 3B). It provided evidence that bilirubin activated the 5-HT_3*A*_ receptor directly, and the normalized intensity of inward currents induced by bilirubin was approximately equivalent to 40% inward currents induced by mCPBG (Figure 3C). By radioligand binding assay, the dose-response curves (Figure 3D and Supplementary Table 3) showed that 5-HT (positive control) had a strong affinity for the 5-HT_3*A*_ receptor, and that the affinity of 8-OH-DPAT (negative control) for the 5-HT_3*A*_ receptor was rather low. Nevertheless, the affinity of bilirubin for the 5-HT_3*A*_ receptor fell in between 5-HT and 8-OH-DPAT, suggesting that bilirubin interacted with the 5-HT_3*A*_ receptor via weak combination bonds rather than ionic or covalent bonds. Moreover, GDCA also had a rather low affinity for the 5-HT_3*A*_ receptor. Homologous modeling and molecular docking were used to predict the possible combination modes of bilirubin and the 5-HT_3*A*_ receptor. Bilirubin entered the hydrophobic gaps formed by the subunits of the 5-HT_3*A*_ receptor with benign complementarity (Figures 4A,B). Calculations showed that bilirubin formed hydrogen bonds with multiple amino acid residues of the 5-HT_3*A*_ receptor. The binding sites were MET661, LYS 119, TYR118, ILE86, TYR38, and ASN103. Hydrogen bonds were formed between TYR38, LYS119, and two carbonyl groups of bilirubin (Figure 4C).

### The 5-HT_3*A*_ Receptor Could Regulate Pain Thresholds Through GABA Pathway in Cholestasis Rats

**FIGURE 5:**
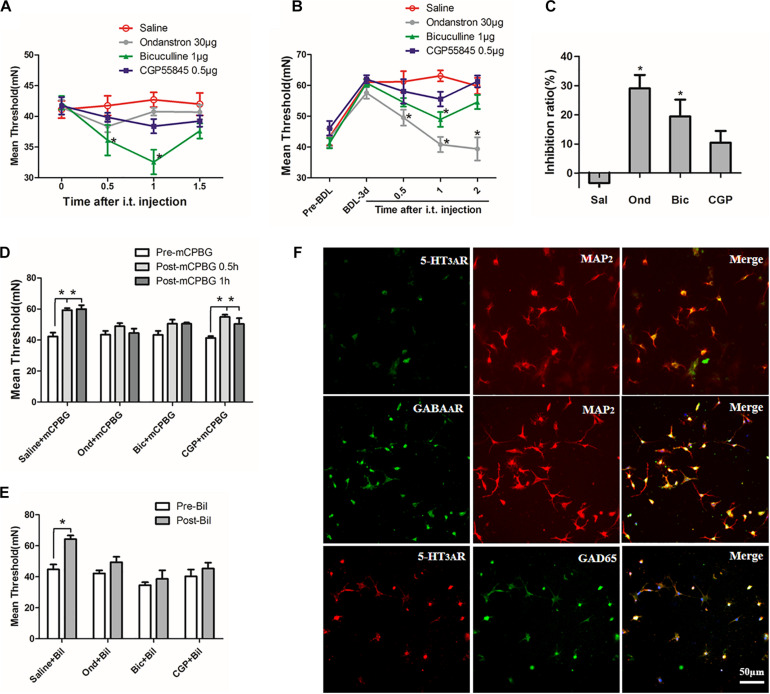
The 5-HT_3*A*_ receptor regulated pain thresholds through GABA pathway in cholestasis rats. **(A)** In normal rats, bicuculline reduced mechanical threshold significantly by intrathecal injection 30 min later. **(B)** In BDL rats, both ondansetron and bicuculline reversed the increased pain thresholds. **(C)** Comparison of inhibition ratios among three antagonists. Results showed that ondansetron exerted stronger inhibition ratio than bicuculline and CGP55845 in BDL rats. **(D)** Both ondansetron and bicuculline, but not CGP55845, could reverse the up-regulation of pain threshold induced by mCPBG in normal rats. **(E)** Ondansetron, bicuculline, and CGP55845 could reverse the effect induced by intrathecally administrating bilirubin in normal rats. **(F)** Expression of the 5-HT_3*A*_ receptor on GABAergic neurons in cultured SDHNs. Most of the cultured neurons (MAP2 is regarded as neuron marker) expressed the 5-HT_3*A*_ receptor and GAD65 (marker of GABAergic neuron). The merged images showed co-expression of the 5-HT_3*A*_ receptor and GAD65, suggesting that the majority of GABAergic neurons expressed the 5-HT_3*A*_ receptor in SDH. *P < 0.05.

To explore the potential mechanism of regulating pain thresholds by the 5-HT_3*A*_ receptor, ondansetron (5-HT_3*A*_ receptor antagonist), bicuculline (GABA_*A*_ receptor antagonist), and CGP55845 (GABA_*B*_ receptor antagonist) were intrathecally administrated into normal or BDL rats, respectively. In normal rats, bicuculline but not CGP55845 could significantly decrease the mechanical threshold (Figure 5A). In BDL rats, both ondansetron and bicuculline could reverse the up-regulation of pain thresholds while CGP55845 had no effects on it (Figure 5B). Moreover, ondansetron exerted stronger inhibition ratio than bicuculline while no difference was observed between CGP55845 and saline in BDL rats (Figure 5C). These results suggested that GABA_*A*_ receptor rather than GABA_*B*_ receptor participated in the regulation of pain thresholds.

Then, mCPBG (5-HT_3*A*_ receptor agonist), ondansetron, CGP55845, and bicuculline were used to further detect the interaction between the 5-HT_3*A*_ receptor and GABA_*A*_ receptor. Results showed that both ondansetron and bicuculline, but not CGP55845, could reverse the effect induced by mCPBG in normal rats (Figure 5D). Similarly, ondansetron, bicuculline, and CGP55845 could also reverse the effect induced by intrathecally administrating bilirubin in normal rats (Figure 5E). These results suggested that bilirubin could increase pain thresholds by activating the 5-HT_3*A*_ receptor and GABA_*A*_ pathway. Further, immunofluorescence on SDHNs showed that the majority of cultured neurons [microtubule-associated protein 2 (MAP2) was considered as neuron marker] expressed 5-HT_3*A*_ receptor and glutamic acid decarboxylase (GAD) 65 (marker of GABAergic neuron). Merged images showed co-expression of the 5-HT_3*A*_ receptor and GAD65, suggesting that most GABAergic neurons expressed the 5-HT_3*A*_ receptor in the SDH (Figure 5F).

### Bilirubin Increased GABA_*A*_ Receptor Expression and GABA Concentration of CSF

**FIGURE 6:**
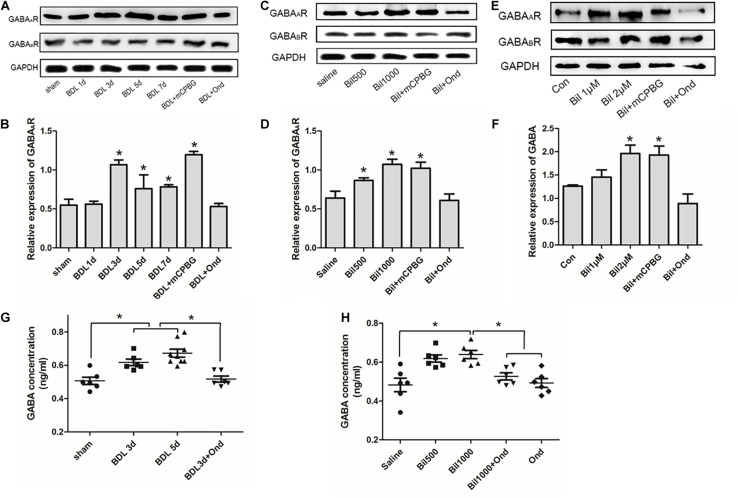
Bilirubin increased GABA_*A*_ receptor expression, GABA concentration, and GABAergic sIPSC in spinal cords. **(A)** GABA_*A*_ receptor expression increased gradually in the spinal cord enlargement of BDL rats without changing GABA_*B*_ receptor expression, and ondansetron reduced GABA_*A*_ receptor expression. **(B)** The quantitative analysis of GABA_*A*_ receptor expression in the BDL rats. **(C)** Intrathecal administration of bilirubin increased GABA_*A*_ receptor expression, and co-administration of bilirubin and ondansetron decreased it without changing GABA_*B*_ receptor expression. **(D)** The quantitative analysis of GABA_*A*_ receptor expression after intrathecal administration of bilirubin in normal rats. **(E)** SDHNs cultured with bilirubin showed increased GABA_*A*_ receptor expression without changing GABA_*B*_ receptor expression, ondansetron reversed GABA_*A*_ receptor expression. **(F)** The quantitative analysis of GABA_*A*_ receptor expression in SDHNs. **(G)** GABA concentration in CSF of BDL group was significantly higher than sham group, and ondansetron intervention reversed this change. **(H)** Intrathecal administration of bilirubin (1000 μM) increased GABA concentration in CSF significantly, whereas ondansetron could reverse it. *P < 0.05.

Results indicated that GABA_*A*_ receptor expression in the spinal cord enlargement of BDL rats increased gradually and was reduced by ondansetron, whereas GABA_*B*_ receptor expression did not change significantly (Figures 6A,B). Intrathecal administration of bilirubin also increased GABA_*A*_ receptor expression, and co-administration of ondansetron blocked the effect of bilirubin without changing GABA_*B*_ receptor expression (Figures 6C,D). Similarly, SDHNs cultured with different concentrations of bilirubin also increased GABA_*A*_ receptor expression, whereas ondansetron reversed it (Figures 6E,F). Moreover, results showed that GABA concentrations in CSF were significantly higher in BDL group (Figure 6G) and in the intrathecally administered bilirubin (1000 μM) group (Figure 6H) than that in control group, and ondansetron could reverse the increase of GABA concentrations.

### Bilirubin Increased the Activity of sIPSC of GABAergic Neurons

**FIGURE 7:**
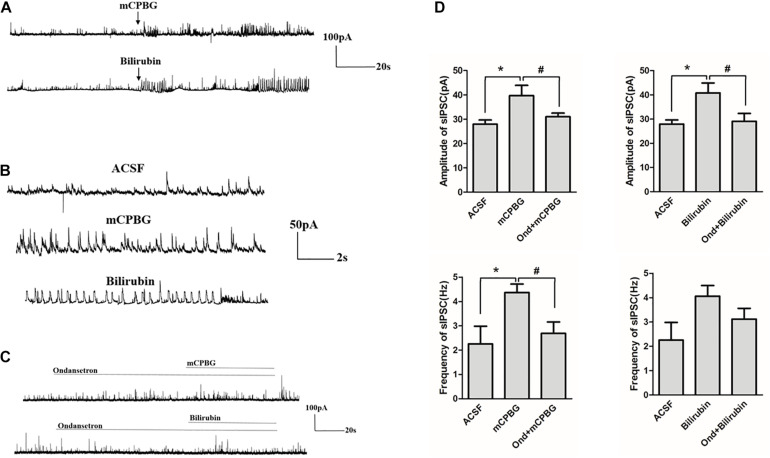
Bilirubin increased the activity of sIPSC of GABAergic neurons. **(A)** Both 0.2 μM of mCPBG and 10 μM of bilirubin enhanced the activity of GABAergic sIPSC significantly. **(B)** The enlargement of currents by ACSF, mCPBG, and bilirubin perfusion. **(C)** No differences were observed by mCPBG or bilirubin intervention in the presence of ondansetron perfusion in GABAergic sIPSC. **(D)** Histogram of the frequency and amplitude of GABAergic sIPSC changed by mCPBG and bilirubin with or without ondansetron intervention. *P < 0.05; ^#^*P* < 0.05.

**FIGURE 8:**
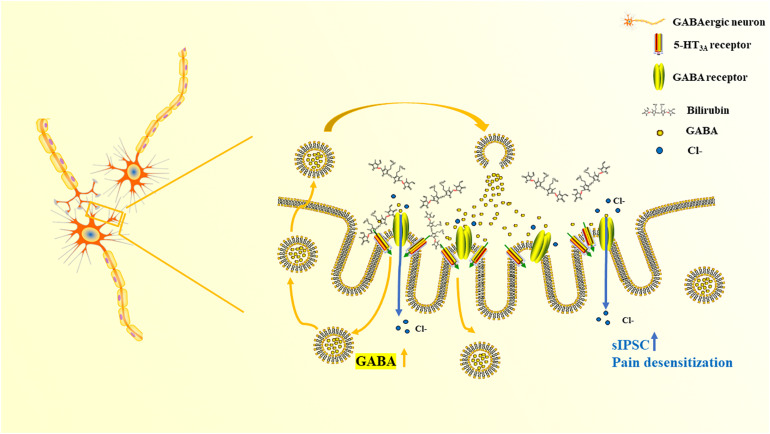
The mechanism map of pain desensitization induced by bilirubin in cholestasis.

Both mCPBG (0.2 μM) and bilirubin (10 μM) enhanced the amplitude of sIPSC of GABAergic neurons significantly (Figures 7A,B). No differences were observed by mCPBG or bilirubin intervention in the presence of ondansetron perfusion in GABAergic sIPSC (Figure 7C). In addition, mCPBG could increase the frequency of sIPSC, whereas bilirubin had no effect on it (Figure 7D). These results suggested that by activating the 5-HT_3*A*_ receptor, bilirubin might influence the amplitude of sIPSC of GABAergic neurons. The mechanism map showed the potential interactions among bilirubin, the 5-HT_3*A*_ receptor, the GABA receptor, and GABA, as shown in our studies (Figure 8).

## Discussion

Cholestasis, characterized by obstruction of intrahepatic and extrahepatic bile ducts, can damage liver and kidney functions because of the accumulation of bilirubin, bile acid, opioids, and other substances (Wang et al., 2013). Cholestasis patients tend to be insensitive to stimulation, as well as showing concealment of symptoms, recovery delay, and pain desensitization. Morphine consumption in cholestasis decreases remarkably by 33% (Tao et al., 2014). Although studies have shown that bile acids can affect the pain sensation by activating peripheral G protein-coupled bile acid receptor 1 (Dawson and Karpen, 2014), it is difficult for bile acids to pass through the BBB. However, as a small molecule, unconjugated bilirubin can pass the BBB readily (Palmela et al., 2012) and be toxic to gliocyte and neurons. Bilirubin can also induce inflammation, neurodegeneration, and apoptosis, which can affect cognition, memory, pain sensation, and even cause kernicterus in newborns (Erlinger et al., 2014). In our study, we found that pain thresholds increased significantly in BDL rats and normal rats which were administered bilirubin intrathecally, suggesting that bilirubin may contribute to pain sensitization in cholestasis.

As the main relay station, SDH regulates the integration of pain signals with various interneurons, and transmits signals to the brain (Gupta and Deshpande, 2008). As the major inhibitory system involved in pain regulation in SDH, the GABAergic pathway plays important roles in neuropathic pain and other types of pain sensation (Kami et al., 2016). GABAergic neurons take up extracellular glutamate and convert it to GABA by GAD. In synaptic clefts, GABA not only inhibits the release of excitatory neurotransmitters directly by activating presynaptic GABA receptors but also induces inhibitory postsynaptic currents by acting on postsynaptic receptors (Tremblay et al., 2016).

As members of the 5-HT receptors family, the 5-HT_3_ receptors are the only non-selective ligand-gated ion channels and are distributed mainly in the cerebral cortex, hippocampus, and amygdala (Wang et al., 2017). An electrical stimulus on the periaqueductal gray can activate 5-HT neurons in the rostral ventromedial medulla and inhibit the excitability of pain-sensitive interneurons, which can be antagonized by 5-HT_3_ receptors antagonists in the spinal cord (Peng et al., 1996). It suggested that the 5-HT_3_ receptors in the spinal cord was involved in regulating interneuron activities. In the spinal cord, 5-HT_3_ receptors are expressed mainly in the postsynaptic membrane of GABAergic neurons in laminae I and II (Lee et al., 2010) and can mediate GABA release in a calcium-dependent manner or enhance sIPSC (Pandhare et al., 2017). Therefore, the 5-HT_3_ receptors exerted important roles in the integration of pain signals.

Morphological studies have shown that most GABAergic neurons in the SDH can express the 5-HT_3*A*_ receptor (Huang et al., 2008). Kawamata et al. (2003) found that intrathecal administration of 5-HT_3*A*_ receptor agonists could increase GABA release while other neurotransmitters such as glutamate and glycine did not change. It suggested that the 5-HT_3*A*_ receptor was associated with GABAergic activities. According to the latest studies, two potential pathways involved were as follows: (1) By activating the 5-HT_3*A*_ receptor in primary afferent nerves, excitatory neurotransmitters are released and act on GABAergic neurons to strength GABA synthesis in the spinal cord (Huang et al., 2008). (2) The 5-HT_3*A*_ receptor could enhance the activities of GABAergic and glycinergic neurons by activating capsaicin receptors and inducing glutamate release in interneurons (Nakatsuka and Gu, 2001). We noticed the response discrepancy to CGP55845 between mCPBG injected group and bilirubin injected group in Figures 4D,E. mCPBG is the specific 5-HT_3*A*_ receptor agonist, while the specific role of bilirubin on 5-HT_3*A*_ receptor is unclear. In Figure 4D, mCPBG intrathecally induced significant increase of pain threshold in the presence of CGP55845, suggesting inhibition of GABA_*B*_ receptor showed little effect on mCPBG-mediated 5-HT_3*A*_ pathway, and GABA_*A*_ receptor may be the vital downstream molecule. In Figure 4E, bilirubin intrathecally did not induce significant increase of pain threshold in the presence of CGP55845, suggesting inhibition of GABA_*B*_ receptor showed effect on bilirubin-mediated 5-HT_3*A*_ pathway, and GABA_*A*_ and GABA_*B*_ receptor both participated in the signal pathway of bilirubin. In our study, we found that the activation of the 5-HT_3*A*_ receptor induced by bilirubin could excite the activity of GABAergic neurons, and it might attribute to the increase of GABA in CSF. Some limitations still need improvement in our study. First, the interaction between bilirubin and GABA receptors was unclear, and further studies were needed to explore if bilirubin could bind with GABA receptors. Second, mechanisms of the increase of GABA in CSF were unclear. The activation of the 5-HT_3*A*_ receptor induced by bilirubin might influence the generation, release, and degradation of GABA in GABAergic neurons. More studies are needed to detect relevant pathways in the future.

## Conclusion

Our study found that elevated bilirubin in cholestasis can enhance the activity of the GABAergic pathway by binding with 5-HT_3*A*_ receptors in SDH. It increased the understandings of the pain desensitization phenomenon in cholestasis and supplied new targets for clinical treatment.

## Data Availability Statement

The original contributions presented in the study are included in the article/Supplementary Material, further inquiries can be directed to the corresponding author/s.

## Ethics Statement

The animal study was reviewed and approved by the Ethics Committee of the Naval Medical University.

## Author Contributions

YS and FW: conceptualization and methodology. XW and XF: software. FW: validation and supervision, project administration, and funding acquisition. EK, HW, and XW: formal analysis and writing—original draft preparation. YZ: investigation. JZ: resources. WY: data curation. YS, FW, and XW: writing—review and editing. EK and XF: visualization. All authors contributed to the article and approved the submitted version.

## Conflict of Interest

The authors declare that the research was conducted in the absence of any commercial or financial relationships that could be construed as a potential conflict of interest.
