# Supplementary material for: Bilirubin Induces Pain Desensitization in Cholestasis by Activating 5-Hydroxytryptamine 3A Receptor in Spinal Cord
Source: Front Cell Dev Biol. 2021 Apr 1;9:605855. doi: 10.3389/fcell.2021.605855 (PMC8047141; doi:10.3389/fcell.2021.605855)
Supplement: Supplementary file 1 [file Table_1.DOCX]

**Supplementary Table 1.** Changes in serum components after bile duct ligation (BDL)

|  | **TB (μmol/L)** | **DB (μmol/L)** | **IB (μmol/L)** | **TBAs (μmol/L)** | **ALT (U/L)** | **AST (U/L)** |
| --- | --- | --- | --- | --- | --- | --- |
| **Sham** | **0.73±0.13** | **0.53±0.11** | **0.2±0.07** | **8.25±0.83** | **54.25±3.34** | **131.5±18.85** |
| **BDL day-1** | **39.73±6.52*** | **37.83±6.63*** | **1.9±0.64** | **178±12.5*** | **401±32.03*** | **1193.5±115.21*** |
| **BDL day-3** | **90.15±11*** | **77.05±8.03*** | **13.1±3.09*** | **117.18±8.06*** | **256±27.87*** | **645.75±81.91*** |
| **BDL day-5** | **66.7±14.3*** | **56.8±11.86*** | **9.9±3.03*** | **87.73±10*** | **112.25±17.98*** | **444±42.5*** |
| **BDL day-7** | **93.82±6.65*** | **79.8±6.6*** | **14.03±1.88*** | **104.43±4.81*** | **119.5±13.29*** | **486.25±21.78*** |

Serum levels of alanine aminotransferase (ALT), aspartate aminotransferase (AST), total bilirubin (TB), direct bilirubin (DB), indirect bilirubin (IB), and total bile acids (TBAs) increased significantly in a time-dependent manner. **P*< 0.05

**Supplementary Table 2.** Changes in cerebrospinal fluid (CSF) components after BDL

|  | **TB**  **(μmol/L)** | **DB**  **(μmol/L)** | **IB**  **(μmol/L)** | **TBA**  **(μmol/L)** | **ALT**  **(U/L)** | **AST**  **(U/L)** |
| --- | --- | --- | --- | --- | --- | --- |
| **Sham** | **0.25±0.05** | **0.13±0.04** | **0.13±0.04** | **0.15±0.05** | **1.25±0.43** | **22.75±9.36** |
| **BDL day-1** | **0.7±0.25** | **0.13±0.04** | **0.58±0.23** | **0.125±0.04** | **1.25±0.43** | **20.25±6.18** |
| **BDL day-3** | **1.3±0.32*** | **0.15±0.05** | **1.15±0.3*** | **0.2±0.07** | **1.75±0.83** | **18.25±9.65** |
| **BDL day-5** | **1.03±0.29*** | **0.13±0.04** | **0.9±0.3*** | **0.175±0.08** | **1.75±0.83** | **17±3.94** |
| **BDL day-7** | **1.13±0.11*** | **0.13±0.04** | **1±0.14*** | **0.2±0.12** | **2.25±1.3** | **28±10.12** |

Only TB and IB in CSF increased significantly 3 days after BDL and no changes were found in TBAs, DB, ALT, or AST. **P*<0.05

**Supplementary Table 3.** Dose-response curve of radioligand binding assay

|  | **5-HT** | **Bilirubin** | **GDCA** | **8-OH-DPAT** |
| --- | --- | --- | --- | --- |
| **Bottom** | **7.089** | **-1.388** | **-2.813** | **-1.599** |
| **Top** | **100** | **100** | **100** | **100** |
| **LogIC_50_** | **2.217** | **4.092** | **5.977** | **4.401** |
| **HillSlope** | **0.9417** | **0.7315** | **0.4736** | **0.5856** |
| **IC_50_** | **164.7** | **12357** | **948109** | **29198** |
